# Synthesis and Anticancer Activity of Novel 9-*O*-Substituted Berberine Derivatives

**DOI:** 10.3390/ijms20092169

**Published:** 2019-05-01

**Authors:** Viktor Milata, Alexandra Svedova, Zuzana Barbierikova, Eva Holubkova, Ingrid Cipakova, Dana Cholujova, Jana Jakubikova, Miroslav Panik, Sona Jantova, Vlasta Brezova, Lubos Cipak

**Affiliations:** 1 Institute of Organic Chemistry, Catalysis and Petrochemistry, Faculty of Chemical and Food Technology, Slovak University of Technology, Radlinskeho 9, 812 37 Bratislava, Slovakia; viktor.milata@stuba.sk (V.M.); evaholubicka@gmail.com (E.H.); 2 Institute of Biochemistry and Microbiology, Faculty of Chemical and Food Technology, Slovak University of Technology, Radlinskeho 9, 812 37 Bratislava, Slovakia; s.svedova@gmail.com (A.S.); sona.jantova@stuba.sk (S.J.); 3 Institute of Physical Chemistry and Chemical Physics, Faculty of Chemical and Food Technology, Slovak University of Technology in Bratislava, Radlinskeho 9, 812 37 Bratislava, Slovakia; zuzana.barbierikova@stuba.sk; 4 Cancer Research Institute, Biomedical Research Center, University Science Park for Biomedicine, Slovak Academy of Sciences, Dubravska cesta 9, 845 05 Bratislava, Slovakia; ingrid.cipakova@savba.sk (I.C.); dana.cholujova@savba.sk (D.C.); jana.jakubikova@savba.sk (J.J.); 5 Institute of Management, Slovak University of Technology, 812 33 Bratislava, Slovakia; miroslav.panik@stuba.sk

**Keywords:** berberine, berberine derivatives, EPR spectroscopy, antitumor activity, cell cycle arrest, apoptosis

## Abstract

Berberine is a bioactive isoquinoline alkaloid derived from many plants. Although berberine has been shown to inhibit growth and induce apoptosis of several tumor cell lines, its poor absorption and moderate activity hamper its full therapeutic potential. Here, we describe the synthesis of a series of 9-*O*-substituted berberine derivatives with improved antiproliferative and apoptosis-inducing activities. An analysis of novel berberine derivatives by EPR spectroscopy confirmed their similar photosensitivity and analogous behavior upon UVA irradiation as berberine, supporting their potential to generate ROS. Improved antitumor activity of novel berberine derivatives was revealed by MTT assay, by flow cytometry and by detection of apoptotic DNA fragmentation and caspase-3 activation, respectively. We showed that novel berberine derivatives are potent inhibitors of growth of HeLa and HL-60 tumor cell lines with IC_50_ values ranging from 0.7 to 16.7 µM for HL-60 cells and 36 to >200 µM for HeLa cells after 48 h treatment. Further cell cycle analysis showed that the observed inhibition of growth of HL-60 cells treated with berberine derivatives was due to arresting these cells in the G_2_/M and S phases. Most strikingly, we found that berberine derivative **3** (9-(3-bromopropoxy)-10-methoxy-5,6-dihydro-[1,3]dioxolo[4,5-g]isoquino[3,2-a] isoquinolin-7-ylium bromide) possesses 30-fold superior antiproliferative activity with an IC_50_ value of 0.7 µM and 6-fold higher apoptosis-inducing activity in HL-60 leukemia cells compared to berberine. Therefore, further studies are merited of the antitumor activity in leukemia cells of this berberine derivative.

## 1. Introduction

Berberine is a well-known bioactive isoquinoline alkaloid present in a wide variety of medicinal plants such as *Berberis aristata*, *B. aquifolium*, *B. vulgaris*, *Hydrastis canadensis*, *Coptis chinensis* and many others [1,2,3]. It has been shown to be active in several diseases such as diabetes, diarrhea, hormonal disorder, coronary heart disease, obesity or hyperlipidemia [2]. Berberine has also shown promising antiprotozoal, antifungal and antimicrobial activities with a range of bacteria [4,5,6]. Furthermore, recent studies have shown that berberine exerts potent anticancer activity towards a variety of cancer cell types, such as colorectal, leukemia, prostate, lung, glioma, esophageal, and ovarian [7,8,9,10,11,12,13].

Detailed studies have shown that berberine can induce apoptosis of tumor cells through its modulation of activity of several pro-apoptotic and anti-apoptotic genes. For example, it alters the Bcl-2/Bax ratio and decreases the mitochondrial membrane potential of selected tumor cells [14]. In addition, it might induce apoptosis through the mitochondrial/caspase pathway, activate caspase-3, and caspase-8, and release the cytochrome *c* [3,8]. It has been also shown that berberine has a variety of effects on the cell cycle and its lower concentrations arrest tumor cells in the G_1_ phase [15,16]. In contrast, higher concentrations of berberine induce the arrest of tumor cells in the G_2_/M phase [17]. Interestingly, it has been reported that berberine might also induce apoptosis of tumor cells by a mechanism that involves the production of a reactive oxygen species (ROS) [18,19,20,21]. Most importantly, several reports have demonstrated that berberine could be combined with radiotherapy or chemotherapy drugs to neutralize their toxicity and enhance their therapeutic activities, thereby improving therapeutic outcomes [22,23,24,25,26,27,28].

Several studies have shown that berberine selectively inhibits tumor cell growth and induces apoptosis while eliciting less cytotoxic effects in normal cells [29,30]. Its poor absorption and moderate activity, however, hamper its full antitumor therapeutic potential [31]. As a result, many laboratories have focused their efforts towards the design and synthesis of novel berberine derivatives with improved biological activities, which has led to the synthesis of numerous berberine derivatives with various modifications at the C8, C9, C10, C12 and C13 positions of the berberine structure [32,33,34,35,36,37,38]. These studies have shown that modifications of berberine at C8 or C13 positions mainly increase its antimicrobial activity, which is closely related to the length of the substituent chains. On the other hand, the modifications to the C9 position of berberine usually increased its antitumor activities [39].

Over the past years, several novel 9-*O*-substituted berberine derivatives, including the 9-*O*-pyrazole alkyl group [40], 9-*O*-chlorohexyl group [41], or 9-*O*-alkyl- and 9-*O*-terpenyl group [35] modified berberine derivatives with improved aqueous solubility and enhanced absorption have been synthesized and tested for their anticancer activity. These studies have shown berberine derivatives to be more potent antitumor agents with improved apoptosis-inducing activity than that of berberine. Additionally, it has been shown that the substitution of berberine on C9 with lipophilic group substitutes, such as ibuprofen and naproxen [42], or cinnamic acid [36], significantly increased the anti-inflammatory and enhanced the hypoglycemic activity of berberine, respectively. These studies point out that modification of berberine on the C9 position can significantly improve its biological activities. Several recent reviews have summarized and outlined in more detail the most important findings on the relationship between various structural modifications of berberine and its improved pharmacological activities [38,43,44].

**Figure 1:**
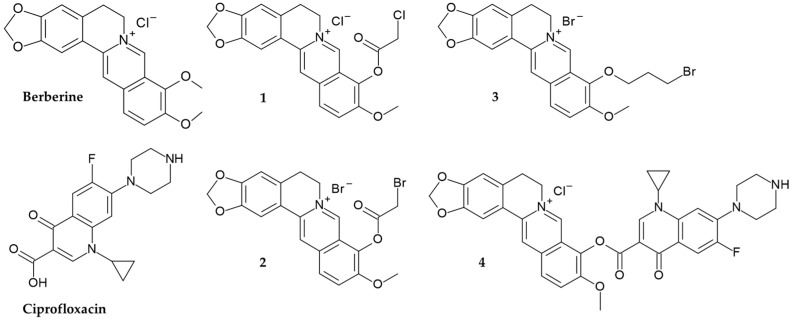
Chemical structures of berberine, ciprofloxacin and newly synthesized berberine derivatives.

**Scheme 1:**
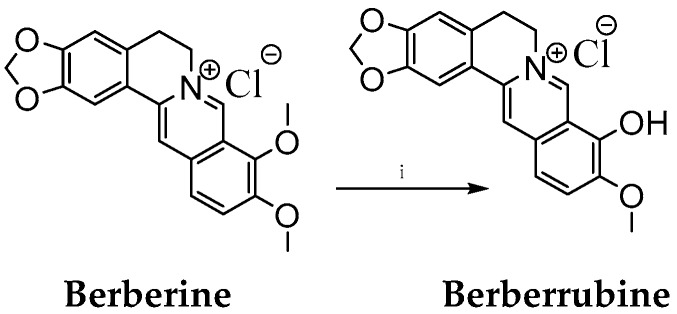
Synthesis of berberrubine. Reaction conditions: (i) 195 °C (temperature of oil bath), 40 min.

**Scheme 2:**
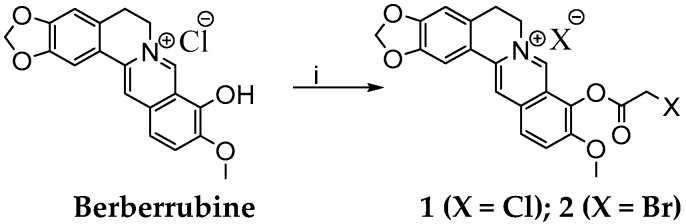
Synthesis of berberine derivative **1** and derivative **2**. Reagents and conditions: (i) CH_3_CN, 40 °C, 15 min, C_2_H_2_Cl_2_O/C_2_H_2_Br_2_O, 2 h.

**Scheme 3:**
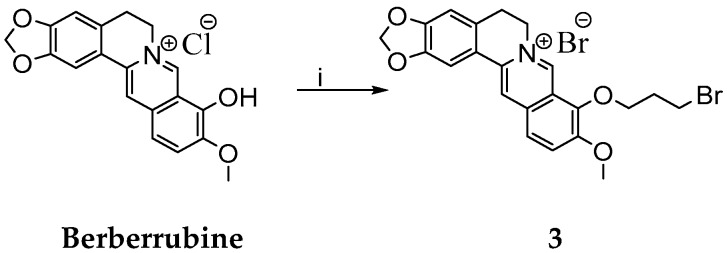
Synthesis of berberine derivative **3**. Reagents and conditions: (i) C_3_H_6_Br_2_, DMF, 80 °C, 2 h.

**Scheme 4:**
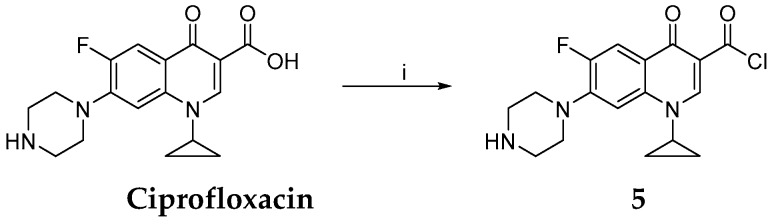
Synthesis of chlorinated ciprofloxacin **5**. Reagents and conditions: (i) SOCl_2_, RT, 2 h.

**Scheme 5:**
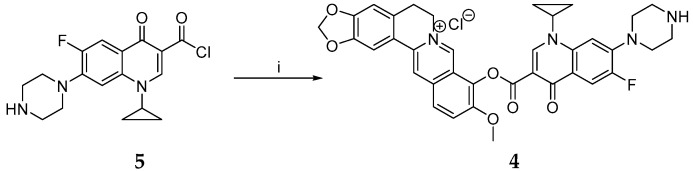
Synthesis of berberine derivative **4**. Reagents and conditions: (i) berberrubine, NaH, dioxane, 300 W, 100 °C, 10 min.

Here, we report the synthesis and evaluation of biological activity of four novel 9-*O*-substituted berberine derivatives. To improve the pharmacological properties and increase the therapeutic efficiency of berberine, we synthesized its 9-*O*-substituted derivatives bearing 2-chloro- and 2-bromo-acetoxy substituents (derivate **1** and **2**), 3-bromopropoxy substituent (derivate **3**) and ciprofloxacin (derivate **4**) (Figure 1, Scheme 1, Scheme 2, Scheme 3, Scheme 4 and Scheme 5). Ciprofloxacin, as a quinolone antibiotic, was selected for the synthesis of berberine derivative **4** as it was reported to have high intrinsic lipophilicity [45] and various biological activity, including the activity against multidrug resistant bacteria *Klebsiella pneumoniae* [46,47].

We demonstrate that novel berberine derivatives maintain similar photosensitivity and analogous behavior upon UVA irradiation to that of berberine, supporting their potential to induce apoptosis through generation of the reactive oxygen species (ROS). We show that novel berberine derivatives are more potent antiproliferative and apoptosis-inducing agents compared to berberine. Moreover, these derivatives arrest HL-60 leukemia cells in the G_2_/M and S phases, with the most promising derivative **3** showing superior apoptosis-inducing activity.

## 2. Results and Discussion

Berberine molecule offers a variety of possible modifications at positions C2, C3, C8, C9, C10, C12, C13 or C=N^+^ [32,33,34,35,36,37,38,48]. Based on previous reports that derivatization of berberine at the C9 position usually increases its antitumor activities [39], we prepared a set of novel 9-*O*-substituted berberine derivatives. Following the established procedures of heating the berberine to 190 °C for at least 15 min under vacuum [35] or under microwave irradiation [49,50], we performed selective demethylation of the berberine on its methoxy group at position C9 to obtain berberrubine (Scheme 1, see section Materials and Methods).

Berberrubine was then treated with 1,3-dibromopropane (DBP) to prepare the berberine derivative **3** (Scheme 3). No base was used here to avoid cyclisation to nitrogen. Various conditions for the synthesis of berberine derivative **3** were tested and obtained yields were summarized in Table 1.

As presented, we found that the high excess of alkylating agent DBP was the most important factor in increasing the yield of the reaction. Similar to the alkylation of berberrubine by DBP, the excess of the haloacetylhalogenides was limiting factor for derivatization of berberrubine to haloacetylesters **1** and **2** (Scheme 2). It should be noted that, under these conditions, the separation of berberine derivatives from reactions was simple because the products were soluble in polar aprotic solvents, thus distinguishing them from the berberrubine. Concerning the synthesis of the berberine-ciprofloxacin conjugate (referred as berberine derivative **4**), the first step of its synthesis was the derivatization of ciprofloxacin to its acylated form (Scheme 4) [51,52]. Prepared carbonyl chloride of ciprofloxacin was further mixed with berberrubine and the reaction was microwave irradiated to produce the berberine derivative **4** in 31% yield (Scheme 5). Importantly, no berberine derivative **4** was obtained without microwave irradiation. It is widely accepted that microwave irradiation is an unconventional energy source with great potential in synthetic organic chemistry [53]. In this respect, microwave irradiation has been applied with success to synthesize berberine derivatives under mild reaction conditions and with short reaction times [49,50,54].

The synthetic routes and characterization of novel berberine derivatives **1**–**4** by ^1^H NMR and ^13^C NMR spectroscopy are described in detail in the Materials and Methods section (Scheme 2, Scheme 3, Scheme 4 and Scheme 5).

It was previously shown that berberine, like many other bioactive molecules, exhibits sensitivity towards light and its photochemical behavior is strongly affected by the solvent and the presence of molecular oxygen [55]. As a photosensitive agent, berberine was previously studied, and the photoinduced generation of ROS, via both electron and energy transfer mechanisms (Types I and II), was evidenced by EPR spectroscopy and direct detection of singlet oxygen phosphorescence [20,56,57]. The investigation of the cytotoxic/phototoxic effect of berberine on murine fibroblast non-cancer NIH-3T3 and Ehrlich ascites carcinoma (EAC) cells showed a statistically significant degree of UVA induced increase of the cytotoxic effect only in cell line EAC. The cell cycle profile analysis revealed that the DNA damage triggered by berberine in combination with UVA irradiation led to a significant blockage of cells in the S and G_2_/M phases with no sub-G_0_ cell fraction detected [20]. Consequently, to provide a more complex view on the newly prepared berberine derivatives, their ability to produce ROS upon UVA irradiation (λ_max_ = 365 nm) was tested using the in situ EPR spin trapping technique.

**Figure 2:**
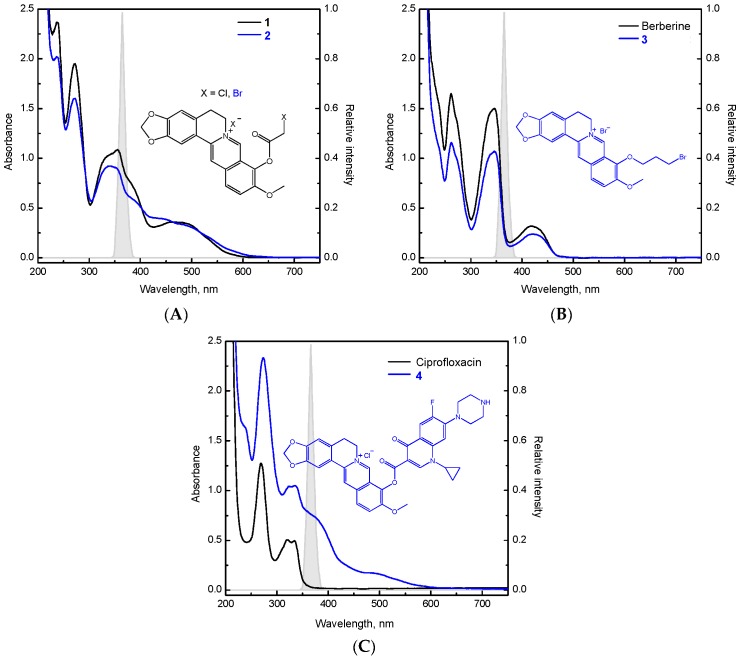
Electronic absorption spectra of (**A**) berberine derivatives **1** and **2**, (**B**) berberine and berberine derivative **3**, (**C**) ciprofloxacin and berberine derivative **4**, all in the mixed solvent DMSO/water (1:100, *v*:*v*; concentration 0.07 mM). Each graph contains the relative spectral distribution of the UVA LED source (λ_max_ = 365 nm) applied in the EPR spin trapping experiments.

The UV/vis absorption spectra of the prepared derivatives were recorded to follow the effect of the structural changes on the absorption maxima (Figure 2). We found that berberine derivative **3** exhibits an analogous spectrum to that of berberine with the characteristic absorption maxima at 348 nm and 421 nm (Figure 2B), while berberine derivatives **1** and **2** reveal a slight bathochromic shift of these bands (355 and 464 nm) together with the presence of other bands in the 300–600 nm region (Figure 2A). The absorption bands reaching higher wavelength up to 600 nm reflect the extended conjugation in berberine derivatives **1** and **2** due to the presence of 1-chloro-acetyl or 1-bromo-acetyl substituents. A combination of berberine with ciprofloxacin (berberine derivative **4**) led to a slight bathochromic shift of the absorption maxima (325 and 337 nm) compared to the ciprofloxacin (321 and 334 nm) and the appearance of broad shoulders at higher wavelength (370 and 490 nm), expanding the absorption more towards the visible region (Figure 2C). As novel berberine derivatives reveal a suitable overlap of their absorption spectra and the emission of UVA light, a source with a maximum at 365 nm was applied in further photochemical experiments.

**Figure 3:**
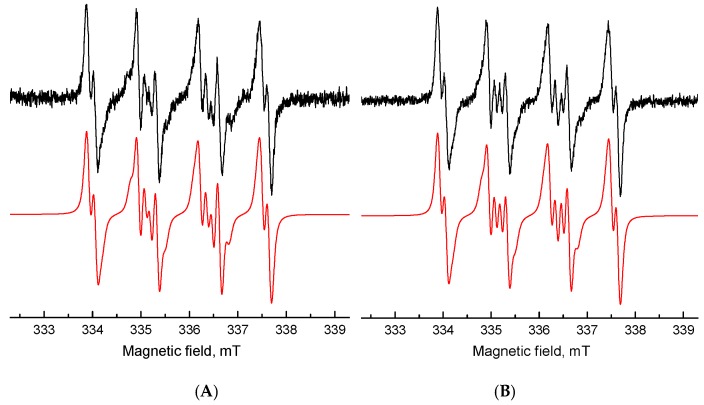
Experimental (**–**) and simulated (**–**) EPR spectra obtained after 13 min UVA exposure of the DMSO solution of (**A**) berberine and (**B**) berberine derivative **3** in the presence of DMPO spin trap under air. (Initial concentrations: c_0_(berberine) = c_0_(berberine derivative **3**) = 0.001 M, c_0_(DMPO) = 0.042 M, λ_max_ = 365 nm, irradiance 13 mW·cm^−2^).

The ROS generation initiated by UVA photoexcitation of berberine derivatives was investigated using indirect techniques of EPR spectroscopy. The application of spin trapping agent 5,5-dimethyl-1-pyrroline *N*-oxide (DMPO) enabled us to follow the formation of reactive radical intermediates upon in situ photoexcitation of berberine derivatives as the corresponding spin-adducts. The EPR spin trapping experiments confirmed that berberine derivatives preserved the photosensitivity of the parent berberine molecule, since they exhibited the photoinduced formation of DMPO spin-adducts upon UVA irradiation, reaching the highest EPR signal intensity for derivative **3** (Figure 3).

The dominant twelve-line signal corresponding to berberine derivative **3** in the spectrum was assigned to the ^•^DMPO-O_2_^−^ (spin-Hamiltonian parameters, *a*_N_ = 1.279 mT, *a*_H_^β^ = 1.025 mT; *a*_H_^γ^ = 0.137 mT; *g* = 2.0059) based on the simulation analysis. This evidences the effective electron transfer from the photoexcited berberine derivative **3** to the molecular oxygen generating the superoxide radical anion stabilized in the aprotic DMSO solvent. Further spin-adduct recognizable in the spectrum belongs to the spin-adduct ^•^DMPO-OCH_3_ (*a*_N_ = 1.303 mT, *a*_H_^β^ = 0.842 mT; *a*_H_^γ^ = 0.147 mT; *g* = 2.0059) originating from the ^•^OCH_3_ produced by the interaction of O_2_^•−^ with DMSO. Even though the absorption of the berberine derivatives is shifted towards the visible region, only a negligible concentration of DMPO spin-adducts upon the visible-light irradiation (*λ* > 420 nm) was found.

**Figure 4:**
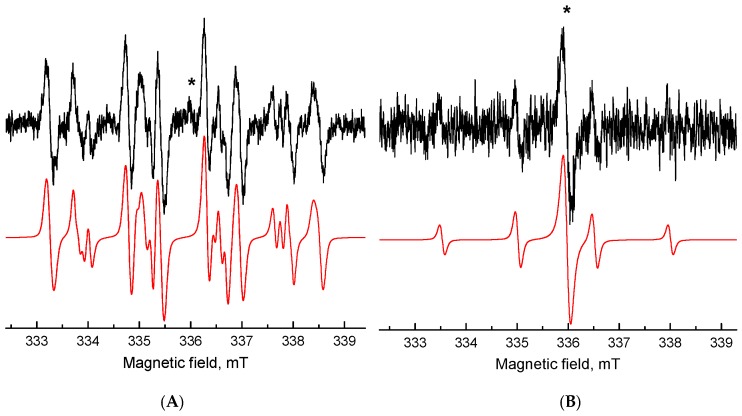
Experimental (**–**) and simulated (**–**) EPR spectra obtained after 13 min UVA exposure of berberine derivative **2** in the presence of DMPO spin trap under air in: (**A**) water/DMSO (2:3, *v*:*v*) and (**B**) water. (Initial concentrations: *c*_0_(berberine derivative **2**) = 0.8 mM, *c*_0_(DMPO) = 0.042 M, *λ*_max_ = 365 nm, irradiance 13 mW·cm^−2^).

The EPR spectra monitored during the continuous irradiation of the berberine and its derivatives **1**–**4** in a mixed water/DMSO (2:3, *v*:*v*) solvent containing DMPO are more complex and represent superposition of signals attributable to four individual DMPO spin-adducts, as shown in the berberine derivative **2** in Figure 4A. Based on the spin-Hamiltonian parameters elucidated from the simulation analysis, the signals were assigned to the spin-adducts ^∙^DMPO-O_2_^−^/O_2_H (*a*_N_ = 1.340 mT, *a*_H_^β^ = 1.069 mT; *a*_H_^γ^ = 0.131 mT; *g* = 2.0059) and ^∙^DMPO-OH (*a*_N_ = 1.433 mT, *a*_H_^β^ = 1.303 mT; *a*_H_^γ^ = 0.071 mT; *g* = 2.0057) [58] and to the two spin-adducts of the carbon-centered radicals with slightly different parameters: ^∙^DMPO-CR1 (*a*_N_ = 1.551 mT, *a*_H_^β^ = 2.197 mT; *g* = 2.0056) and ^∙^DMPO-CR2 (*a*_N_ = 1.506 mT, *a*_H_^β^ = 2.121 mT; *g* = 2.0057). The last two spin-adducts were produced via interaction of photogenerated ROS with DMSO or berberine molecules. Moreover, a low-intensity single-line signal (*g* ~ 2.0043, asterisk in Figure 4A) with unresolved hyperfine structure was found upon UVA photoexcitation of the berberine derivative **2** in water/DMSO (2:3, *v*:*v*) solution under given experimental conditions, which was most probably produced by the electron transfer reactions of the photoexcited berberine derivative **2**, and its *g*-value is compatible with the oxygen-centered radicals [59]. The generation of this radical species was observed also upon continuous UVA irradiation of the aqueous solution of berberine derivative **2** in the presence of DMPO spin trap, along with the low-intensity four-line signal of ^∙^DMPO-OH spin-adduct (*a*_N_ = 1.506 mT, *a*_H_^β^ = 1.478 mT; *g* = 2.0057) (Figure 4B).

Upon the continuous UVA irradiation of berberine derivatives **1**–**4** in the presence of sterically-hindered amine 4-oxo-2,2,6,6-tetramethylpiperidine (TMPO) in DMSO, we only monitored the generation of low-intensity EPR signal of 4-oxo-2,2,6,6-tetramethylpiperidine *N*-oxyl (Tempone) produced via TMPO oxidation by singlet oxygen. The generated nitroxide radical Tempone is most likely transformed to the diamagnetic products by the interaction of >NO^•^ moiety with other radical intermediates [60], effectively formed upon the UVA photoexcitation of berberine derivatives **1**–**4**.

Overall, the EPR spin trapping experiments demonstrated analogous behaviour of novel berberine derivatives upon UVA irradiation involving effective ROS generation to that of berberine. It will be interesting to examine these berberine derivatives for their dose-dependent accumulation of intracellular ROS to find out if generated ROS are involved in their cytotoxicity.

The antiproliferative activity of berberine derivatives was carried out by the 3-(4,5-dimethylthiazol-2-yl)-2,5-diphenyltetrazolium bromide (MTT) assay and rated in half-maximal inhibitory concentration, which is a measure of the effectiveness of an agent in inhibiting the growth and viability of cells [61]. The antiproliferative activity of berberine derivatives in tumor HeLa and HL-60 cell lines is summarized in Table 2.

We observed an increase in antiproliferative activity of berberine derivatives **1**–**4** against tested tumor cell lines compared to berberine or ciprofloxacin. Cytotoxic activity was observed in berberine derivatives **1**, **2** and **4** with IC_50_ values ranging from 95.0 to ˃200 µM for HeLa cells and from 11.2 to 23.4 µM for HL-60 cells. The lowest antiproliferative activity was found for berberine derivative **4** with IC_50_ values ranging from 15.7 to ˃200 µM. Most strikingly, the highest antiproliferative activity towards tested tumor cell lines was observed in berberine derivative **3** with IC_50_ values of 58.0 and 36.0 for HeLa cells and 3.7 and 0.7 µM for HL-60 cells treated for 24 and 48 h, respectively. This finding is in agreement with the previous study showing that 9-*O*-pyrazole alkyl substituted berberine derivatives have increased anticancer activity compared to berberine, with IC_50_ values ranging from 48.8 to 100.4 µM for HeLa cells after 24 h treatment [40].

**Figure 5:**
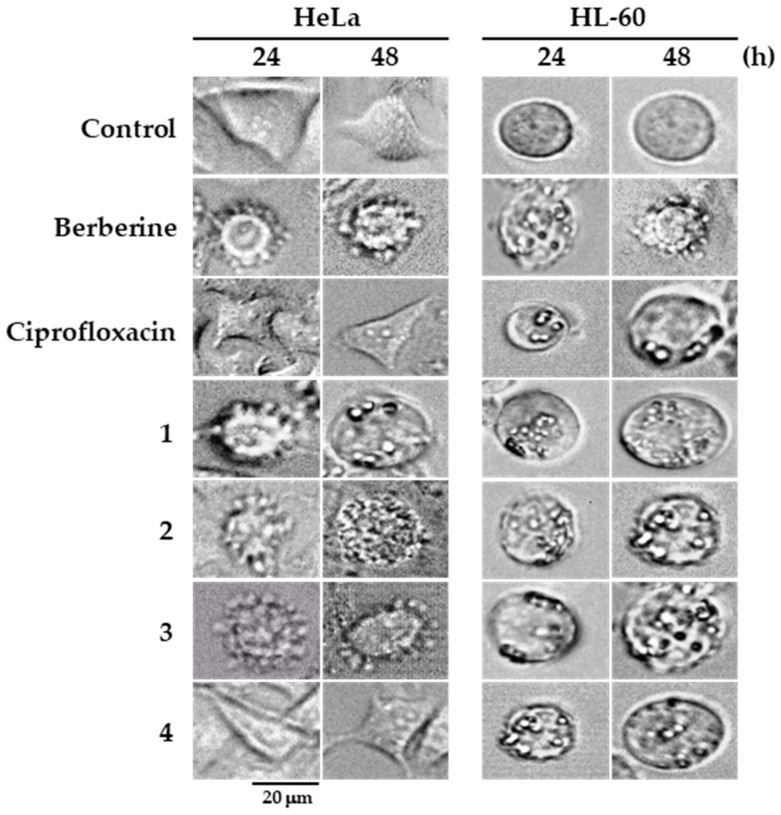
Assessment of changes in cellular morphology of HeLa and HL-60 cells treated with berberine, ciprofloxacin, and berberine derivatives (10 µM) for the time indicated. Magnification of microscope: ×600.

The effect of berberine derivatives towards tumor HeLa and HL-60 cells was further assessed by analyzing their impact on the cellular morphology of cells treated for 24 and 48 h (Figure 5). As shown, untreated HeLa cells had normal epithelial morphology. In contrast, in some tumor cells, exposure to 10 µM concentrations of berberine or its derivatives **1**, **2** and **3** induced the formation of cell membrane blebbing after 24 and 48 h of treatment. However, HeLa cells treated with 10 µM concentrations of ciprofloxacin and berberine derivative **4** did not show any morphological changes. On the other hand, compared to untreated HL-60 cells, which have the rounded morphology characteristic of leukemia cells, the treatment of HL-60 cells with 10 µM concentrations of berberine, ciprofloxacin and berberine derivatives **1**–**4** resulted in profound formation of cell membrane blebs.

**Figure 6:**
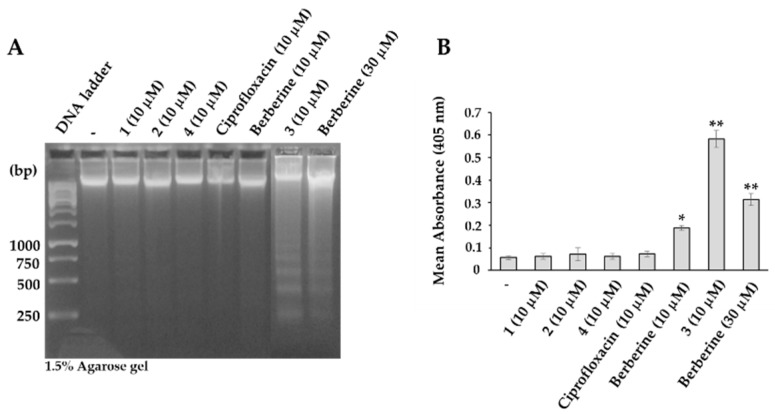
(**A**) Detection of apoptotic DNA fragmentation in HL-60 cells treated with indicated concentrations of berberine, ciprofloxacin and berberine derivatives for 48 h. (**B**) Activation of caspase-3 expressed as the mean absorbance at 405 nm (the relative amount of the pNA released from the DEVD-pNA). Each point represents a mean ± S.D. from 3 independent experiments. * *p* < 0.05, ** *p* < 0.01.

As cell membrane blebbing, observed in sub-population of berberine derivative-treated HeLa and HL-60 cells, is one of the defining features of apoptosis [62,63], next, we analyzed the potential of novel berberine derivatives to induce apoptosis. We compared their ability to induce apoptosis by employing DNA laddering and caspase-3 activation assays. As presented, after incubation of HL-60 cells with 10 µM concentrations of berberine derivatives for 48 h, only berberine derivative **3** treated HL-60 cells were positive for apoptotic DNA fragmentation (Figure 6A) and had active caspase-3 (Figure 6B). Comparing the intensities of apoptotic DNA fragmentation and activation of caspase-3 in HL-60 cells treated with 30 µM berberine and 10 µM berberine derivative **3**, berberine derivative **3** clearly exhibited 30-fold superiority to that of berberine.

**Figure 7:**
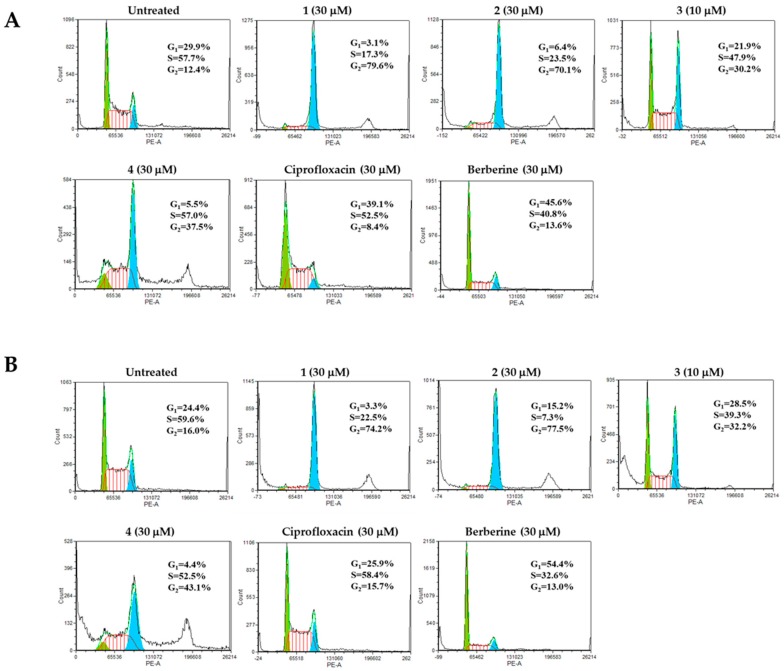
Analysis of cell cycle distribution of HL-60 cells treated with indicated concentrations of berberine, ciprofloxacin and berberine derivatives **1–4** for 24 h (**A**) and 48 h (**B**).

To further extend our cytotoxicity studies, we analyzed the effect of novel berberine derivatives on the cell cycle of HL-60 leukemia cells. We found that in contrast to berberine, which at 30 µM concentrations arrests HL-60 cells in the G_0_/G_1_ phase, the novel berberine derivatives **1** and **2** (30 µM) and **3** (10 µM) arrest HL-60 cells at the G_2_/M phase with a proportional decrease of cells in the G_0_/G_1_ and S phases, while berberine derivative **4** induces G_2_/M and S phases-arrest (Figure 7). 

This finding indicates that novel berberine derivatives are more active molecules than berberine and ciprofloxacin, which in the same concentration have no effect or induce G_0_/G_1_ cell cycle arrest of HL-60 leukemia cells [15,16,17]. The observed G_2_/M cell cycle arrest of HL-60 cells treated with berberine derivatives **1**–**4** supports their higher antiproliferative activity. Interestingly, berberine derivative **3** induced a significant increase in the number of cells defined as the sub-G_0_ cell population (1.8% in untreated vs. 21.9% in berberine derivative **3** treated cells, Figure 7B), confirming its apoptotis-inducing potential.

In conclusion, we have synthesized a series of four novel 9-*O*-substituted berberine derivatives and evaluated their antiproliferative activity in human HeLa and HL-60 tumor cell lines. We found these derivatives to be more active antitumor agents than their parental molecules, berberine and ciprofloxacin. Most importantly, we showed that berberine derivative **3** (9-(3-bromopropoxy)-10-methoxy-5,6-dihydro-[1,3]dioxolo[4,5-g]isoquino[3,2-a]isoquinolin-7-ylium bromide) possesses the highest cytotoxic activity and ability to induce apoptosis of HL-60 leukemia cells. Therefore, this compound could become a promising candidate for anticancer therapy, and has been selected for the follow-up studies to reveal the molecular mechanisms of its antitumor action.

## 3. Materials and Methods

### 3.1. Experimental Methods

#### 3.1.1. Chemistry

##### General

All reagents and solvents were purchased from Sigma-Aldrich^®^ (Darmstadt, Germany), Alfa-Aesar^®^ (Ward Hill, MA, USA), Fluka^®^ (Buchs, Switzerland) and Mikrochem^®^ (Pezinok, Slovakia). Solvents (acetonitrile, DMF, dioxane, THF) were purified and/or dried using standard laboratory methods and stored over molecular sieves (4 Å). Column chromatography was performed using silicagel Nomasil—40–63 μm (VWR^®^, Randor, PA, USA) and displayed eluent. Reaction progress was monitored by thin layer chromatography on Silufol or Alufol plates (Merck^®^, Darmstadt, Germany) with a UV indicator for λ = 254 nm.

Melting points (m. p.) of the prepared compounds were established on a Kofler block using digital thermometer TD 121 (VWR^®^, Randor, PA, USA).

Spectrometers INOVA 300 (300 MHz, 75 MHz, 282 MHz; Varian Inc., Palo Alto, CA, USA), VXR-300, Bruker (300 MHz, 75 MHz, Karlsruhe, Germany), and VNMRS 600 (600 MHz, 151 MHz, 564 MHz; Varian Inc., Palo Alto, CA, USA) were used for measurements of ^1^H, ^13^C and ^19^F NMR spectra, at their frequencies at RT. Chemical shifts in (δ)–[ppm] (parts per million) were referenced to residual signal of the solvent. Coupling constants (J) were given in [Hz] with multiplicity: s (singlet), d (doublet), dd (doublet of the doublet), t (triplet), q (quartet), q (quintet) and m (multiplet).

Spectrometer Nicolet NEXUS 470 FT-IR (Thermo Fisher Scientific^®^, Madison, WI, USA) was used to obtain ATR IR absorption bands (ν) in [cm^−1^]. High resolution mass spectra (HR-MS) were recorded with spectrometer LTQ Orbitrap XL (Thermo Fisher Scientific^®^, Madison, WI, USA). Microwave irradiation was realized in a CEM Discover microwave reactor (CEM Corporation, Matthews, NC, USA).

##### Selective Demethylation of Berberine on Its C9 Position

Berberine (2.0 g, 5.38 mmol) was placed into a round-bottomed flask equipped with a cross head magnetic stirrer. The flask was inserted into an oil bath preheated to 200 °C and attached to a vacuum pump. The berberine was then intensively stirred at 195 °C for 40 min (Scheme 1). After that, the obtained berberrubine was crystallized using a mixture of ethanol:methanol (1:1) by refluxing for 10 min. The obtained crystals of berberrubine were filtered using a Büchner funnel, washed with ethanol, and vacuum dried in a water bath at 40 °C.

The yield of berberrubine was 1.25 g (62.5%, red-brownish powder, m. p.: 275–279 °C).

**^1^H NMR:** (CD_3_OD) *δ* 9.28 (s, 1H, H-8), 8.70 (s, 1H, H-13), 7.54 (t, 1H, ^3^J_HH_ = 8.04 Hz, H-12), 7.49 (s, 1H, H-1), 6.91 (d, 1H, ^3^J_HH_ = 7.60 Hz, H-11), 6.85 (s, 1H, H-4), 6.04 (s, 2H, H-2’), 4.60 (t, 2H, ^3^J_HH_ = 5.32 Hz, H-6), 3.89 (s, 3H, H-10′), 3.12 (t, 2H, ^3^J_HH_ = 5.46 Hz, H-5).

**^13^C NMR:** (CD_3_OD) *δ* 149.5 (C-13′), 149.4 (C-9), 148.0 (C-10), 145.9 (C-8), 134.2 (C-2), 132.2 (C-3), 129.2 (C-12′), 122.6 (C-8′), 121.4 (C-4′), 120.1 (C-13″), 118.3 (C-12), 107.7 (C-11), 107.1 (C-13), 104.4 (C-4), 101.8 (C-1), 56.9 (C-2′), 55.2 (C-10′), 54.1 (C-6), 27.5 (C-5).

##### Synthesis of 9-(2-Chloro- or Bromo-acetoxy)-10-metoxy-5,6-dihydro-[1,3]dioxolo[4,5-g]isoquino[3,2-a] isoquinoline-7-ylium Chloride or Bromide

Berberrubine (0.7 g, 1.96 mmol) was dissolved in dry CH_3_CN and stirred at 40 °C for 15 min. After that, haloacetylhalides chloracetyl chloride (0.29 mL, 2.55 mmol) or bromacetyl bromide (0.22 mL, 2.55 mmol) were dropped into the stirred reaction mixture, followed by stirring for additional 2 h. After cooling the mixture to RT, the products were filtered off, washed with dry CH_3_CN and left to dry in a vacuum.

The yield of berberine derivative **1** was 0.42 g (49%, yellow powder, m. p. > 300 °C).

**^1^H NMR:** (CD_3_OD) *δ* 10.03 (s, 1H, H-8), 8.72 (s, 1H, H-13), 8.54 (d, 1H, ^3^J_HH_ = 8.05 Hz, H-11), 8.19 (d, 1H, ^3^J_HH_ = 8.61 Hz, H-12), 7.27 (s, 1H, H-1), 7.16 (s, 1H, H-4), 6.34 (s, 2H, H-2′), 5.12 (t, 2H, ^3^J_HH_ = 5.55 Hz, H-6), 4.19 (s, 2H, H-9″), 3.78 (s, 3H, H-10′), 3.14 (t, 2H, ^3^J_HH_ = 5.34 Hz, H-5).

**^13^C NMR:** (CD_3_OD) *δ* 159.8 (C-9′), 152.6 (C-10), 147.9 (C-3), 147.1 (C-2), 146.3 (C-8), 140.1 (C-9), 135.5 (C-13′), 132.2 (C-12′), 131.9 (C-4′), 124.4 (C-11), 121.8 (C-12), 120.1 (C-8′), 119.1 (C-13″), 118.2 (C-13), 106.7 (C-4), 105.1 (C-1), 100.3 (C-2′), 61.1 (C-10′), 53.8 (C-6), 44.7 (C-9″) 21.77 (C-5).

The yield of berberine derivative **2** was 0.38 g (38%, yellow-orange powder, m. p. > 300 °C).

**^1^H NMR:** (CD_3_OD) *δ* 9.83 (s, 1H, H-8), 8.71 (s, 1H, H-13), 8.64 (d, 1H, ^3^J_HH_ = 8.15 Hz, H-11), 7.87 (d, 1H, ^3^J_HH_ = 8.25 Hz, H-12), 7.23 (s, 1H, H-1), 7.14 (s, 1H, H-4), 6.72 (s, 2H, H-2′), 5.13 (t, 2H, ^3^J_HH_ = 5.74 Hz, H-6), 4.22 (s, 2H, H-9″), 3.92 (s, 3H, H-10′), 3.03 (t, 2H, ^3^J_HH_ = 5.21 Hz, H-5).

**^13^C NMR:** (CD_3_OD) *δ* 161.3 (C-9′), 149.2 (C-10), 148.1 (C-3), 145.7(C-2), 143.1 (C-8), 142.6 (C-9), 138.4 (C-13′), 134.8 (C-12′), 131.6 (C-4′), 129.1 (C-11), 123.3 (C-12), 122.7 (C-8′), 120.1 (C-13″), 117.8 (C-13), 109.2 (C-4), 107.4 (C-1), 100.9 (C-2′), 63.1 (C-10′), 52.5 (C-6), 45.9 (C-9″) 23.8 (C-5).

##### Synthesis of 9-(3-Bromopropoxy)-10-methoxy-5,6-dihydro-[1,3]dioxolo[4,5-g]isoquino[3,2-a] isoquinolin-7-ylium Bromide

Berberrubine (0.5 g, 1.40 mmol) was dissolved in dry DMF (10 mL) at 80 °C and stirred for 15 min. Then, 1,3-dibromopropane (8.2 mL, 80.75 mmol) was added dropwise and the reaction was stirred for 2 h. After cooling to RT, the diethyl ether (20 mL) was added. Separated precipitate was filtered off using Büchner funnel. Obtained berberine derivative **3** was further purified using column chromatography (eluent CHCl_3_:MeOH—10:1).

The yield of berberine derivative **3** was 0.54 g (74%, yellow powder, m. p. > 300 °C).

**^1^H NMR:** (CD_3_OD) *δ* 9.79 (s, 1H, H-8), 8.94 (s, 1H, H-13), 8.20 (d, 1H, ^3^J_HH_ = 8.25 Hz, H-11), 8.00 (d, 1H, ^3^J_HH_ = 8.25 Hz, H-12), 7.79 (s, 1H, H-1), 7.09 (s, 1H, H-4), 6.17 (s, 2H, H-2′), 4.94 (t, 2H, ^3^J_HH_ = 5.98 Hz, H-6), 4.42 (t, 2H, ^3^J_HH_ = 9.28 Hz, H-9′), 4.06 (s, 3H, H-10′), 3.94 (t, 2H, H-9″′), 3.21 (t, 2H, ^3^J_HH_ = 5.58 Hz, H-5), 2.33 (p, 2H, H-9″).

**^13^C NMR:** (CD_3_OD) *δ* 150.2 (C-10), 149.8 (C-3), 147.6 (C-2), 145.2 (C-8), 142.4 (C-9), 137.5 (C-13′), 133.0 (C-12′), 130.7 (C-4′), 126.7 (C-11), 123.5 (C-12), 121.4 (C-8′), 120.4 (C-13″), 120.2 (C-13), 108.4 (C-4), 105.4 (C-1), 102.0 (C-2′), 71.2 (C-9′), 57.1 (C-10′), 55.2 (C-6), 42.1 (C-9″′), 32.6 (C-9″), 26.3 (C-5).

##### Synthesis of 1-Cyclopropyl-6-fluoro-4-oxo-7-piperazin-1-yl-1,4-dihydroquinoline-3-carbonyl Chloride

A round bottomed flask (equipped with Dimroth condenser and calcium chloride tube) containing thionyl chloride (0.85 mL, 11.7 mmol) was cooled in ice bath and the apparatus was filled with argon. After cooling off the flask content, ciprofloxacin (0.1 g, 0.3 mmol) was added. The colour of the suspension turned orange. The sample was stirred for 2 h. During stirring, the temperature was allowed to reach RT. Then dry CH_2_Cl_2_ (20 mL) was slowly added and the suspension was allowed to exsiccate until dry using a rotary vacuum evaporator. The procedure was repeated 3 times to remove the excess of HCl and thionyl chloride. As the result, the target carbonyl chloride derived from ciprofloxacin **5** was obtained [42].

The yield of chlorinated ciprofloxacin **5** was 0.085 g (81%, red powder, m. p. 246–247 °C).

**^1^H NMR:** (TFA-d) *δ* 9.63 (s, 1H, H-2), 9.47 (d, 1H, ^3^J_HF_ = 12.92 Hz, H-5), 8.13 (d, 1H, ^4^J_HF_ = 5.36 Hz, H-8), 3.93–4.49 (m, 5H, H-10, H-14, H-1′), 3.13 (t, 4H, ^3^J_HH_ = 4.71 Hz, H-11, H-13), 2.43 (s, 1H, H-12), 1.47–1.85 (m, 4H, H-1′, H-1″).

**^13^C NMR:** (TFA-d) *δ* 178.2 (C-4), 162.3 (C-3′), 155.4 (C-2), 149.9 (C-6), 149.5 (C-7), 148.1 (C-8′), 117.2 (C-3), 115.1 (C-4′), 112.8 (C-5), 107.6 (C-8), 52.1, 52.3 (C-10, C-14), 44.6, 44.9 (C-11, C-13), 32.6 (C-1′), 12.6, 12.8 (C-1″, C-1″′).

**^19^F NMR:** (TFA-d) *δ* −77.95 (F-6).

##### Synthesis of 9-(1-Cyclopropyl-6-fluoro-4-oxo-7-piperazin-1-yl-1,4-dihydroquinoline-3-carbonyloxy)-10-methoxy-5,6-dihydro-[1,3]dioxolo[4,5-g]isoquino[3,2-a]isoquinolin-7-ylium Chloride

During continuous stirring, berberrubine (0.071 g, 0.19 mmol) and NaH (0.03 g, 1.14 mmol) and after 15 min chlorinated ciprofloxacin **5** (0.07 g, 0.19 mmol) were slowly added to dry dioxane (5 mL). The reaction mixture was then irradiated for 10 min using 300 W power at a temperature of 100 °C. After cooling, the mixture was exsiccated until dry and further purified using column chromatography on silicagel using MeOH:CHCl_3_ (1:10) as an eluent.

The yield of berberrubine-ciprofloxacin conjugate, also referred to as berberine derivative **4** was 0.039 g (31%, dark brown powder, m. p. > 300 °C).

**^1^H NMR:** (TFA-d) *δ* 9.43 (s, 1H, H-8), 9.31 (s, 1H, H-2C), 8.98 (d, 1H, ^3^J_HF_ = 13.83 Hz, C-5C), 8.47 (d, 1H, ^3^J_HF_ = 5.15 Hz, H-8C), 8.27 (d, 1H, ^3^J_HH_ = 8.73 Hz, H-11), 7.98–8.12 (m, 3H, H-1, H-4, H-12), 6.63 (s, 2H, H-2′), 5.28 (t, 2H, ^3^J_HH_ = 6.02 Hz, H-6), 4.25 (s, 3H, H-10′), 3.25–4.18 (m, 11H, H-5, H-10C, H-11C, H-13C, H-14C, H-1′C), 2.98 (s, 1H, H-12C), 1.53–1.72 (m, 4H, H-1″C, H-1″′C).

**^13^C NMR:** (TFA-d) *δ* 176.1 (C-4C), 167.5 (C-3′C), 159.7 (C-2C), 152.6 (C-10), 151.7 (C-6C), 150.3 (C-7C), 148.1 (C-3), 146.9 (C-2), 144.9 (C-8), 143.5, 144.1 (C-9, C-8′), 140.0 (C-13′), 135.1 (C-12′), 131.7 (C-4′), 123.8 (C-11), 123.1 (C-12), 119.3, 120.7, 121.9 (C-8′, C-13″, C-3C), 118.5 (C-13), 115.4 (C-8C), 106.3, 106.9, 108.2 (C-1, C-4, C-8C), 100.1 (C-2′), 62.1 (C-10′), 53.8, 54.6, 55.1 (C-6, C-10C, C-14C), 48.7, 49.3 (C-11, C-13), 38.7 (C-1′C), 31.4 (C-5), 14.7, 14.9 (C-1″, C-1″′).

**^19^F NMR:** (TFA-d) *δ* −75.71 (F6).

**HRMS (ESI)**: calculated for C_36_H_32_ClFN_4_O_6_ (M + H^+^): 671.1994; found: 670.9106.

#### 3.1.2. Electron Paramagnetic Resonance (EPR) Spectroscopy Studies of Berberine Derivatives

The UV/visible spectra of berberine, ciprofloxacin and newly synthesized berberine derivatives dissolved in the mixed solvent DMSO/water (1:100, *v*:*v*) were recorded using a UV-3600 UV-vis-NIR spectrophotometer (Shimadzu, Kyoto, Japan) with a 1-cm square quartz cell.

The formation of paramagnetic intermediates of the studied berberine derivatives upon their UVA irradiation was monitored in situ using the EPR spin trapping technique. The photoinduced production of singlet oxygen during the excitation of berberine derivatives was followed by the oxidation of a sterically-hindered amine 4-oxo-2,2,6,6-tetramethylpiperidine (TMPO). The solution of the berberine derivatives was mixed either with the spin trapping agent 5,5-dimethyl-1-pyrroline N-oxide (DMPO) or TMPO solution, and immediately after a careful air or argon saturation using a slight gas stream, the prepared sample was transferred to a small quartz flat cell (WG 808-Q, optical cell length 0.04 cm; Wilmad-LabGlass, Vineland, NJ, USA) optimized for the TE102 cavity (Bruker, Rheinstetten, Germany) of the X-band EPR spectrometer (EMX Plus, Bruker, Rheinstetten, Germany). The photoexcitation of the samples took place directly in the EPR resonator at 295 K and the EPR spectra were recorded in situ during the continuous irradiation or after a defined exposure. A UV LED monochromatic radiator (λ_max_ = 365 nm; Bluepoint LED, Hönle UV Technology, Gräfelfing/München, Germany) reaching the irradiance value of 13 mW·cm^−2^ within the EPR cavity, determined using a UVX radiometer (UVP, Upland, CA, USA) or the visible-light source (KL 1600LED (T = 5600 K; Schott)) were used as the irradiation sources. The *g*-values were determined using a built-in nuclear magnetic resonance teslameter (ER 036TM, Bruker) and an integrated frequency counter. The EPR spectra were analyzed and simulated using Bruker software WinEPR and EasySpin toolbox working under Matlab software (MathWorks, Natick, MA, USA) [64].

#### 3.1.3. Evaluation of Antiproliferative Activities of Berberine Derivatives

##### Cell Cultures and Determination of Cell Viability and Morphology

HL-60 (human promyelocytic leukemia) and HeLa (human cervix epithelioid carcinoma) cells were grown in a humidified atmosphere containing 5% CO_2_ and 95% air at 37 °C. HeLa cells were maintained in a DMEM medium and HL-60 cells were maintained in RPMI 1640 medium, both supplemented with 10% (*v*/*v*) heat-inactivated fetal bovine serum (FBS), 50 U/mL penicillin, and 50 µg/mL streptomycin. Stock solutions of berberine and its derivatives were prepared in DMSO, except for a stock solution of ciprofloxacin, which was prepared in 0.1 M HCl. In all experiments, the final concentration of DMSO was kept ≤ 0.1% (*v*/*v*) and 0.1% DMSO alone was used as vehicle control. Cell viability was determined using a 3-(4,5-dimethylthiazol-2-yl)-2, 5-diphenyltetrazolium bromide (MTT) assay [61]. HL-60 cells (2 × 10^4^ cells/well) or HeLa cells (4 × 10^4^ cells/well) were seeded in 96-well plates. Cells were treated with various concentrations of berberine, ciprofloxacin and berberine derivatives (0–100 µM) for 24 and 48 h. At the end of the treatment, MTT solution (final concentration 1 mg/mL) was added to each well and the plates were incubated for another 4 h. The precipitated formazan was dissolved in DMSO and its concentration was measured at 595 nm using a microplate reader (xMark, BioRad, Tokyo, Japan). Based on the curve fitting using nonlinear regression (Origin 7.0, Microcal), the median inhibitory concentrations IC_50_ values (the concentration resulting in 50% inhibition of the cell proliferation that was recorded in control experiments) were determined separately for each experiment. The values were calculated from three independent experiments. Data are presented as a mean ± SD (*n* = 3).

The morphology of cells treated with tested drugs was evaluated using light microscopy (Zeiss Jenalumar, Jena, Germany) [65].

##### Detection of Induction of Apoptosis by DNA Laddering Assay

Cells (2 × 10^6^) treated with various concentrations of tested derivatives were washed with ice-cold PBS containing 10 mM EDTA. Pellets of cells were resuspended in 250 µL of lysis buffer (10 mM Tris-HCl, pH 8.0, 100 mM NaCl, 5 mM EDTA, 5% Triton-X, 0.25% SDS) supplemented with 0.4 mg/mL DNase-free RNase A, incubated at 65 °C for 15 min, cooled to RT for 10 min, and incubated at 37 °C for 90 min. After that, Proteinase K was added to a final concentration of 0.2 mg/mL and samples were incubated at 50 °C for an additional 1 h. The DNA was extracted with 200 µL of phenol:chloroform:isoamyl alcohol (25:24:1) for 1 min and centrifuged at 14,500× *g* for 5 min. The DNA was precipitated from the aqueous phase with 3 volumes of chilled EtOH containing 0.3 M sodium acetate. The DNA pellets were washed with 70% EtOH, dried, and dissolved in 100 µL of TE buffer. The DNA was separated on 1.5% agarose gel and visualized under UV illumination after being stained with GelRed Nucleic Acid Gel Stain (254 nm, Ultra-Lum Electronic UV Transilluminator, Paramount, CA, USA) [66].

##### Detection of Apoptosis by Caspase-3 Assay

Activation of caspase-3 was measured using a colorimetric caspase-3 assay kit (CaspACE^TM^ Assay System, Promega, Madison, WI, USA). Briefly, exponentially growing HL-60 cells were treated with tested derivatives (10–30 µM) for 48 h. Cells (5 × 10^6^) were pelleted by centrifugation (1200× *g*, 3 min at 4 °C) and resuspended in 50 μL of lysis buffer (Promega, Madison, WI, USA). The lysed cell mixture was then incubated on ice for 15 min before centrifugation (13,000× *g*, 5 min at 4 °C). 42 μL of reaction buffer supplemented with 10 mM DTT were mixed with 20 µL of cell lysates. The substrate DEVD-pNA (2 µL) was added and the samples were incubated for 4 h at 37 °C. The release of *p*-nitroaniline from specific caspase-3 substrate was measured at 405 nm using a microplate reader (xMark, BioRad, Tokyo, Japan).

##### Determination of Cell Cycle Distribution

Cells (0.5 × 10^6^) treated with berberine derivatives (0–30 µM) for 24 and 48 h were washed with PBS and fixed in ice-cold 70% ethanol. Next, the cells were washed twice with PBS and resuspended in 0.1% Triton-X in PBS containing 50 µg/mL RNase A and incubated for 30 min at 37 °C. Afterwards, DNA was stained using PI (50 µg/mL) for 15 min at 4 °C. The cell cycle distribution was analyzed using a FACS Canto II flow cytometer (Becton Dickinson, Franklin Lakes, NJ, USA). A minimum of 10,000 cells per sample was analyzed at a flow rate of 200 cells/s [67].

## Figures and Tables

**Table 1:** Reaction conditions for the preparation of berberine derivative **3** (1 equivalent of substrate, no base used).

Reaction	DBP (Equivalents)	Solvent	Temperature (°C)	Time (h)	Yield (%)
R_1_	3	CH_3_CN	60	2	48
R_2_	10	CH_3_CN	60	2	55
R_3_	10	DMF	80	2	59
R_4_	58	DMF	80	2	74

**Table 2:** IC_50_ values (µM) for antiproliferative activity of berberine, ciprofloxacin and berberine derivatives **1**–**4** determined by MTT assay.

Cell Line	Time (h)	Berberine	Ciprofloxacin	1	2	3	4
HeLa	24	˃200	˃200	˃200	˃200	58 ± 2.6	˃200
	48	126 ± 7.8	175 ± 8.7	115 ± 7.4	95 ± 4.8	36 ± 1.9	˃200
HL-60	24	43.7 ± 1.1	˃200	23.4 ± 2.6	14.6 ± 1.7	2.7 ± 0.9	19.3 ± 1.3
	48	27.1 ± 2.6	142 ± 5.7	16.7 ± 0.8	11.2 ± 1.6	0.7 ± 0.2	15.7 ± 1.9

## Abbreviations

DBP	1,3-Dibromopropane
DMF	Dimethylformamide
DMPO	5,5-Dimethyl-1-pyrroline *N*-oxide
DMSO	Dimethyl Sulfoxide
DTT	Dithiothreitol
MTT	3-(4,5-Dimethylthiazol-2-yl)-2,5-diphenyltetrazolium bromide
PBS	Phosphate buffer saline
PI	Propidium iodide
ROS	Reactive Oxygen Species
RT	Room temperature
Tempone	4-Oxo-2,2,6,6-tetramethylpiperidine *N*-oxyl
THF	Tetrahydrofuran
TMPO	4-Oxo-2,2,6,6-tetramethylpiperidine
